# Astaxanthin: Sources, Extraction, Stability, Biological Activities and Its Commercial Applications—A Review

**DOI:** 10.3390/md12010128

**Published:** 2014-01-07

**Authors:** Ranga Rao Ambati, Phang Siew Moi, Sarada Ravi, Ravishankar Gokare Aswathanarayana

**Affiliations:** 1 Institute of Ocean and Earth Sciences, University of Malaya, Kuala Lumpur 50603, Malaysia; E-Mail: phang@um.edu.my; 2 Plant Cell Biotechnology Department, Central Food Technological Research Institute, (Constituent Laboratory of Council of Scientific & Industrial Research), Mysore-570020, Karnataka, India; E-Mail: sarada_ravi@yahoo.com; 3 C. D. Sagar Centre for Life Sciences, Dayananda Sagar Institutions, Kumaraswamy Layout, Bangalore-560078, Karnataka, India; E-Mail: rgokare@yahoo.co.in

**Keywords:** astaxanthin, sources, stability, biological activities, health benefits, applications

## Abstract

There is currently much interest in biological active compounds derived from natural resources, especially compounds that can efficiently act on molecular targets, which are involved in various diseases. Astaxanthin (3,3′-dihydroxy-β, β′-carotene-4,4′-dione) is a xanthophyll carotenoid, contained in *Haematococcus pluvialis*, *Chlorella zofingiensis*, *Chlorococcum*, and *Phaffia rhodozyma*. It accumulates up to 3.8% on the dry weight basis in *H. pluvialis.* Our recent published data on astaxanthin extraction, analysis, stability studies, and its biological activities results were added to this review paper. Based on our results and current literature, astaxanthin showed potential biological activity in *in vitro* and *in vivo* models. These studies emphasize the influence of astaxanthin and its beneficial effects on the metabolism in animals and humans. Bioavailability of astaxanthin in animals was enhanced after feeding *Haematococcus* biomass as a source of astaxanthin. Astaxanthin, used as a nutritional supplement, antioxidant and anticancer agent, prevents diabetes, cardiovascular diseases, and neurodegenerative disorders, and also stimulates immunization. Astaxanthin products are used for commercial applications in the dosage forms as tablets, capsules, syrups, oils, soft gels, creams, biomass and granulated powders. Astaxanthin patent applications are available in food, feed and nutraceutical applications. The current review provides up-to-date information on astaxanthin sources, extraction, analysis, stability, biological activities, health benefits and special attention paid to its commercial applications.

## 1. Introduction

Astaxanthin is a xanthophyll carotenoid which is found in various microorganisms and marine animals [1]. It is a red fat-soluble pigment which does not have pro-Vitamin A activity in the human body, although some of the studies reported that astaxanthin has more potent biological activity than other carotenoids. The United States Food and Drug Administration (USFDA) has approved the use of astaxanthin as food colorant in animal and fish feed [2]. The European Commission considers natural astaxanthin as a food dye [3]. *Haematococcus pluvialis* is a green microalga, which accumulates high astaxanthin content under stress conditions such as high salinity, nitrogen deficiency, high temperature and light [4,5,6]. Astaxanthin produced from *H. pluvialis* is a main source for human consumption [7]. It is used as a source of pigment in the feed for salmon, trout and shrimp [1,3]. For dietary supplement in humans and animals, astaxanthin is obtained from seafood or extracted from *H. pluvialis* [8]*.* The consumption of astaxanthin can prevent or reduce risk of various disorders in humans and animals [7,8]. The effects of astaxanthin on human health nutrition have been published by various authors [7,8,9,10,11,12,13]. In our previous reviews, we included recent findings on the potential effects of astaxanthin and its esters on biological activities [14,15,16,17,18]. The use of astaxanthin as a nutritional supplement has been rapidly growing in foods, feeds, nutraceuticals and pharmaceuticals. This present review paper provides information on astaxanthin sources, extraction methods, storage stability, biological activities, and health benefits for the prevention of various diseases and use in commercial applications.

## 2. Source of Astaxanthin

**Figure 1:**
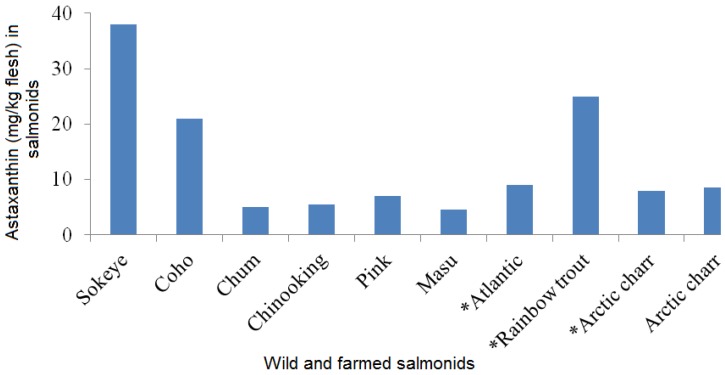
Astaxanthin levels (mg/kg flesh) of wild and farmed (*) salmonids [20].

The natural sources of astaxanthin are algae, yeast, salmon, trout, krill, shrimp and crayfish. Astaxanthin from various microorganism sources are presented in Table 1. The commercial astaxanthin is mainly from *Phaffia* yeast, *Haematococcus* and through chemical synthesis. *Haematococcus pluvialis* is one of the best sources of natural astaxanthin [17,18,19,20]. Astaxanthin content in wild and farmed salmonids are shown in Figure 1. Among the wild salmonids, the maximum astaxanthin content in wild *Oncorhynchus* species was reported in the range of 26–38 mg/kg flesh in sockeye salmon whereas low astaxanthin content was reported in chum [20]. Astaxanthin content in farmed Atlantic salmon was reported as 6–8 mg/kg flesh. Astaxanthin is available in the European (6 mg/kg flesh) and Japanese market (25 mg/kg flesh) from large trout. Shrimp, crab and salmon can serve as dietary sources of astaxanthin [20]. Wild caught salmon is a good source of astaxanthin. In order to get 3.6 mg of astaxanthin one can eat 165 grams of salmon per day. Astaxanthin supplement at 3.6 mg per day can be beneficial to health as reported by Iwamoto *et al.* [21].

**Table 1:** Microorganism sources of astaxanthin.

Sources	Astaxanthin (%) on the Dry Weight Basis	References
**Chlorophyceae**		
*Haematococcus pluvialis*	3.8	[17,18]
*Haematococcus pluvialis* (K-0084)	3.8	[22]
*Haematococcus pluvialis* (Local isolation)	3.6	[23]
*Haematococcus pluvialis* (AQSE002)	3.4	[24]
*Haematococcus pluvialis* (K-0084)	2.7	[25]
*Chlorococcum*	0.2	[26,27]
*Chlorella zofingiensis*	0.001	[28]
*Neochloris wimmeri*	0.6	[29]
**Ulvophyceae**		
*Enteromorpha intestinalis*	0.02	[30]
*Ulva lactuca*	0.01	[30]
**Florideophyceae**		
*Catenella repens*	0.02	[30]
**Alphaproteobacteria**		
*Agrobacterium aurantiacum*	0.01	[31]
*Paracoccus carotinifaciens* (NITE SD 00017)	2.2	[32]
**Tremellomycetes**		
*Xanthophyllomyces dendrorhous* (JH)	0.5	[33]
*Xanthophyllomyces* *dendrorhous* (VKPM Y2476)	0.5	[34]
**Labyrinthulomycetes**		
*Thraustochytrium* sp. CHN-3 (FERM P-18556)	0.2	[35]
**Malacostraca**		
*Pandalus borealis*	0.12	[20]
*Pandalus clarkia*	0.015	[36]

## 3. Structure of Astaxanthin

**Figure 2:**
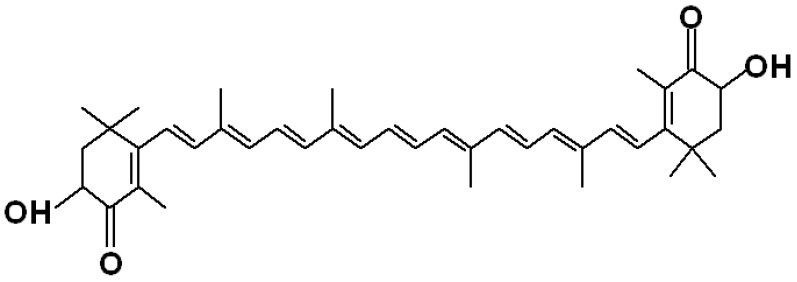
Planner structure of astaxanthin.

**Figure 3:**
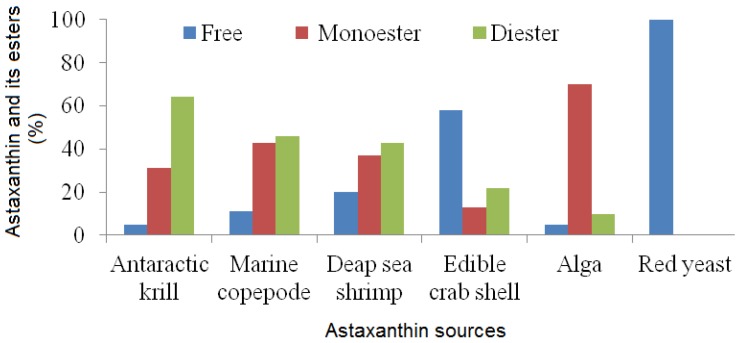
Astaxanthin and its esters from various sources [19,20].

Astaxanthin is a member of the xanthophylls, because it contains not only carbon and hydrogen but also oxygen atoms (Figure 2). Astaxanthin consists of two terminal rings joined by a polyene chain. This molecule has two asymmetric carbons located at the 3, 3′ positions of the β-ionone ring with hydroxyl group (-OH) on either end of the molecule. In case one, hydroxyl group reacts with a fatty acid then it forms mono-ester, whereas when both hydroxyl groups are reacted with fatty acids the result is termed a di-ester. Astaxanthin exists in stereoisomers, geometric isomers, free and esterified forms [1]. All of these forms are found in natural sources. The stereoisomers (3*S*, *3*′*S*) and (3*R* 3′*R*) are the most abundant in nature*. Haematococcus* biosynthesizes the (3*S*, 3′*S*)-isomer whereas yeast *Xanthophyllomyces dendrorhous* produces (3*R*, 3′*R*)-isomer [10]. Synthetic astaxanthin comprises isomers of (3*S*, 3′*S*) (3*R*, 3′*S*) and (3*R*, 3′*R*). The primary stereoisomer of astaxanthin found in the Antarctic krill *Euphausia superba* is 3*R*, 3′*R* which contains mainly esterified form, whereas in wild Atlantic salmon it is 3*S*, 3′*S* which occurs as the free form [37]. The relative percentage of astaxanthin and its esters in krill, copepod, shrimp and shell is shown in Figure 3. Astaxanthin has the molecular formula C_40_H_52_O_4_. Its molar mass is 596.84 g/mol.

## 4. Extraction and Analysis of Astaxanthin

Astaxanthin is a lipophilic compound and can be dissolved in solvents and oils. Solvents, acids, edible oils, microwave assisted and enzymatic methods are used for astaxanthin extraction. Astaxanthin is accumulated in encysted cells of *Haematococcus*. Astaxanthin in *Haematococcus* was extracted with different acid treatments, hydrochloric acid giving up to 80% recovery of the pigment [38]. When encysted cells were treated with 40% acetone at 80 °C for 2 min followed by kitalase, cellulose, abalone and acetone powder, 70% recovery of astaxanthin was obtained [39]. High astaxanthin yield was observed with treatment of hydrochloric acid at various temperatures for 15 and 30 min using sonication [40]. In another study, vegetable oils (soyabean, corn, olive and grape seed) were used to extract astaxanthin from *Haematococcus*. The culture was mixed with oils, and the astaxanthin inside the cell was extracted into the oils, with the highest recovery of 93% with olive oil [41]. Astaxanthin (1.3 mg/g) was extracted from *Phaffia rhodozyma* under acid conditions [42]. Microwave assisted extraction at 75 °C for 5 min resulted in 75% of astaxanthin; however, astaxanthin content was high in acetone extract [43,44]. Astaxanthin yield from *Haematococcus* was 80%–90% using supercritical fluid extraction with ethanol and sunflower oil as co-solvent [45,46,47]. Astaxanthin was extracted repeatedly with solvents, pooled and evaporated by rotary evaporator, then re-dissolved in solvent and absorbance of extract was measured at 476–480 nm to estimate the astaxanthin content [17]. Further the extract can be analyzed for quantification of astaxanthin using high pressure liquid chromatography and identified by mass spectra [18].

## 5. Storage and Stability of Astaxanthin

Astaxanthin stability was assessed in various carriers and storage conditions. Astaxanthin derived from *Haematococcus* and its stability in various edible oils was determined [48]. Astaxanthin was stable at 70–90 °C in ricebran, gingelly and palm oils with 84%–90% of retention of astaxanthin content which can be used in food, pharmaceutical and nutraceutical applications, whereas astaxanthin content was reduced at 120 and 150 °C [48]. Astaxanthin nanodispersions’ stability was evaluated in skimmed milk, orange juice and deionized water was used as a control [49]. It was found that degradation of astaxanthin was significantly higher in skimmed milk than orange juice. In another study, stability of astaxanthin biomass was examined after drying and storage at various conditions for nine weeks [50]. The results showed that degradation of astaxanthin was as low as 10% in biomass dried at 180/110 °C and stored at −21 °C under nitrogen after nine weeks of storage. The stability of astaxanthin from *Phaffia rhodozyma* was studied and it was found that stability was high at pH 4.0 and at a lower temperature [51]. The storage stability of astaxanthin was enhanced at 4 °C and 25 °C in a complex mixture of hydroxyproply-β-cyclodextrin and water [52]. Astaxanthin stability was investigated using microencapsulation with chitosan, polymeric nanospheres, emulsions and β-cyclodextrin as reported by various authors [53,54,55,56].

## 6. Biochemistry of Astaxanthin

**Figure 4:**
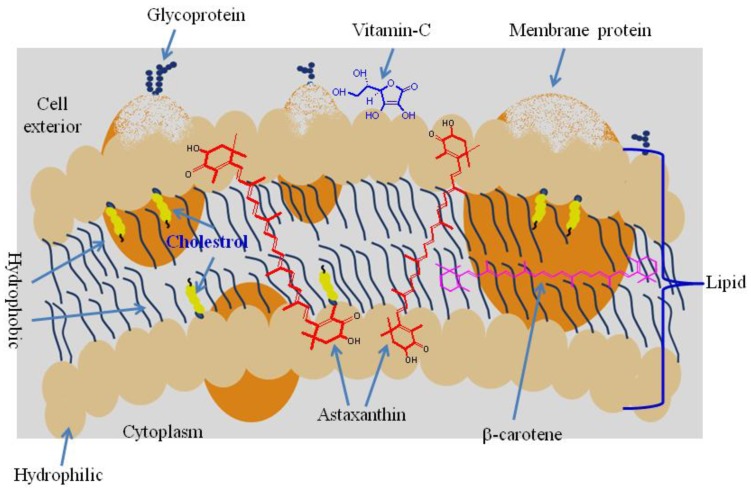
Superior position of astaxanthin in the cell membrane [12].

Astaxanthin contains conjugated double bonds, hydroxyl and keto groups. It has both lipophilic and hydrophilic properties [1]. The red color is due to the conjugated double bonds at the center of the compound. This type of conjugated double bond acts as a strong antioxidant by donating the electrons and reacting with free radicals to convert them to be more stable product and terminate free radical chain reaction in a wide variety of living organisms [8]. Astaxanthin showed better biological activity than other antioxidants [11], because it could link with cell membrane from inside to outside (Figure 4).

## 7. Bioavailability and Pharmacokinetics of Astaxanthin

### 7.1. Bioavailability

Dietary oils may enhance the absorption of astaxanthin. Astaxanthin with combination of fish oil promoted hypolipidemic/hypocholesterolemic effects in plasma and its increased phagocytic activity of activated neutrophils when compared with astaxanthin and fish oil alone [57]. Astaxanthin was superior to fish oil in particular by improving immune response and lowering the risk of vascular and infectious diseases. The proliferation activity of T- and B-lymphocytes was diminished followed by lower levels of O_2_, H_2_O_2_ and NO production, increased antioxidant enzymes superoxide dismutase, catalase and glutathione peroxidase (GPx), and calcium release in cytosol after administration of astaxanthin with fish oil [58]. Bioavailability and antioxidant properties of astaxanthin were enhanced in rat plasma and liver tissues after administration of *Haematococcus* biomass dispersed in olive oil [14,15,17].

Astaxanthin is a fat soluble compound, with increased absorption when consumed with dietary oils. Astaxanthin was shown to significantly influence immune function in several *in vitro* and *in vivo* assays [14,15,17]. Lipophilic compounds such as astaxanthin are usually transformed metabolically before they are excreted, and metabolites of astaxanthin have been detected in various rat tissues [59]. Astaxanthin bioavailability in human plasma was confirmed with single dosage of 100 mg [60]. Its accumulation in humans was found after administration of *Haematococcus* biomass as source of astaxanthin [61]. Astaxanthin bioavailability in humans was enhanced by lipid based formulations; high amounts of carotenes solubilized into the oil phase of the food matrix can lead to greater bioavailability [62]. A recent study reported that astaxanthin accumulation in rat plasma and liver was observed after feeding of *Haematococcus* biomass as source of astaxanthin [14,15,17].

### 7.2. Pharmacokinetics

Carotenoids are absorbed into the body like lipids and transported via the lymphatic system into the liver. The absorption of carotenoids is dependent on the accompanying dietary components. A high cholesterol diet may increase carotenoid absorption while a low fat diet reduces its absorption. Astaxanthin mixes with bile acid after ingestion and make micelles in the intestinum tenue. The micelles with astaxanthin are partially absorbed by intestinal mucosal cells. Intestinal mucosal cells incorporate astaxanthin into chylomicra. Chylomicra with astaxanthin are digested by lipoprotein lipase after releasing into the lymph within the systemic circulation, and chylomicron remnants are rapidly removed by the liver and other tissues. Astaxanthin is assimilated with lipoproteins and transported into the tissues [62]. Of several naturally occurring carotenoids, astaxanthin is considered one of the best carotenoids being able to protect cells, lipids and membrane lipoproteins against oxidative damage.

## 8. Biological Activities of Astaxanthin and Its Health Benefits

### 8.1. Antioxidant Effects

An antioxidant is a molecule which can inhibit oxidation. Oxidative damage is initiated by free radicals and reactive oxygen species (ROS). These molecules have very high reactivity and are produced by normal aerobic metabolism in organisms. Excess oxidative molecules may react with proteins, lipids and DNA through chain reaction, to cause protein and lipid oxidation and DNA damage which are associated with various disorders. This type of oxidative molecules can be inhibited by endogenous and exogenous antioxidants such as carotenoids. Carotenoids contain polyene chain, long conjugated double bonds, which carry out antioxidant activities by quenching singlet oxygen and scavenging radicals to terminate chain reactions. The biological benefits of carotenoids may be due to their antioxidant properties attributed to their physical and chemical interactions with cell membranes. Astaxanthin had higher antioxidant activity when compared to various carotenoids such as lutein, lycopene, α-carotene and β-carotene reported by Naguib *et al.* [63]. The antioxidant enzymes catalase, superoxide dismutase, peroxidase and thiobarbituric acid reactive substances (TBARS) were high in rat plasma and liver after feeding *Haematococcus* biomass as source of astaxanthin [17]. Astaxanthin in *H. pluvialis* offered the best protection from free radicals in rats followed by β-carotene and lutein [15,17]. Astaxanthin contains a unique molecular structure in the presence of hydroxyl and keto moieties on each ionone ring, which are responsible for the high antioxidant properties [10,64]. Antioxidant activity of astaxanthin was 10 times more than zeaxanthin, lutein, canthaxanthin, β-carotene and 100 times higher than α-tocopherol [65]. The oxo functional group in carotenoids has higher antioxidant activity without pro-oxidative contribution [66]. The polyene chain in astaxanthin traps radicals in the cell membrane, while the terminal ring of astaxanthin could scavenge radicals at the outer and inner parts of cell membrane (Figure 4). Antioxidant enzyme activities were evaluated in the serum after astaxanthin was supplemented in the diet of rabbits, showing enhanced activity of superoxide dismutase and thioredoxin reductase whereas paraoxonase was inhibited in the oxidative-induced rabbits [67]. Antioxidant enzyme levels were increased when astaxanthin fed to ethanol-induced gastric ulcer rats [68].

### 8.2. Anti-Lipid Peroxidation Activity

Astaxanthin has a unique molecular structure which enables it to stay both in and outside the cell membrane. It gives better protection than β-carotene and Vitamin C which can be positioned inside the lipid bilayer. It serves as a safeguard against oxidative damage by various mechanisms, like quenching of singlet oxygen; scavenging of radicals to prevent chain reactions; preservation of membrane structure by inhibiting lipid peroxidation; enhancement of immune system function and regulation of gene expression. Astaxanthin and its esters showed 80% anti-lipid peroxidation activity in ethanol induced gastric ulcer rats and skin cancer rats [14,68]. Astaxanthin inhibited lipid peroxidation in biological samples reported by various authors [14,15,17,18,68,69].

### 8.3. Anti-Inflammation

Astaxanthin is a potent antioxidant to terminate the induction of inflammation in biological systems. Astaxanthin acts against inflammation. Algal cell extracts of *Haematococcus* and *Chlorococcum* significantly reduced bacterial load and gastric inflammation in *H. pylori*-infected mice [16,70,71]. Park *et al.* [72] reported astaxanthin reduced the DNA oxidative damage biomarker inflammation, thus enhancing immune response in young healthy adult female human subjects. Haines *et al.* [73] reported lowered bronchoalveolar lavage fluid inflammatory cell numbers, and enhanced cAMP, cGMP levels in lung tissues after feeding astaxanthin with *Ginkgo biloba* extract and Vitamin C. Another study showed astaxanthin esters and total carotenoids from *Haematococcus* exerted a dose-dependent gastroprotective effect on acute, gastric lesions in ethanol-induced gastric ulcers in rats. This may be due to inhibition of H_1_, K_1_ ATPase, upregulation of mucin content and an increase in antioxidant activities [68]. Astaxanthin showed protective effect on high glucose induced oxidative stress, inflammation and apoptosis in proximal tubular epithelial cells. Astaxanthin is a promising molecule for the treatment of ocular inflammation in eyes as reported by the Japanese researchers [74,75]. Astaxanthin can prevent skin thickening and reduce collagen reduction against UV induced skin damage [14,76,77].

### 8.4. Anti-Diabetic Activity

Generally, oxidative stress levels are very high in diabetes mellitus patients. It is induced by hyperglycemia, due to the dysfunction of pancreatic β-cells and tissue damage in patients. Astaxanthin could reduce the oxidative stress caused by hyperglycemia in pancreatic β-cells and also improve glucose and serum insulin levels [78]. Astaxanthin can protect pancreatic β-cells against glucose toxicity. It was also shown to be a good immunological agent in the recovery of lymphocyte dysfunctions associated with diabetic rats [79]. In another study, ameliorate oxidative stress in streptozotocin-diabetes rats were inhibited by the combination of astaxanthin with α-tocopherol [80]. It is also inhibited glycation and glycated protein induced cytotoxicity in human umbilical vein endothelial cells by preventing lipid/protein oxidation [81]. Improved insulin sensitivity in both spontaneously hypertensive corpulent rats and mice on high fat plus high fructose diets was observed after feeding with astaxanthin [82,83,84]. The urinary albumin level in astaxanthin treated diabetic mice was significantly lower than the control group [78]. Some of the studies demonstrated that astaxanthin prevents diabetic nephropathy by reduction of the oxidative stress and renal cell damage [85,86,87].

### 8.5. Cardiovascular Disease Prevention

Astaxanthin is a potent antioxidant with anti-inflammatory activity and its effect examined in both experimental animals and human subjects. Oxidative stress and inflammation are pathophysiological features of atherosclerotic cardiovascular disease. Astaxanthin is a potential therapeutic agent against atherosclerotic cardiovascular disease [88]. The efficacy of disodium disuccinate astaxanthin (DDA) in protecting mycocardium using mycocardial ischemia reperfusion model in animals was evaluated. Myocardial infarct size was reduced in Sprague Dawley rats, and improved in myocardial salvage in rabbits after four days of pre-treatment with DDA at 25, 50 and 75 mg/kg body weight [89,90]. Astaxanthin was found in rat mycocardial tissues after pretreatment with DDA at dosage of 150 and 500 mg/kg/day for seven days [91]. Astaxanthin effects on blood pressure in spontaneously hypertensive rats (SHR), normotensive Wistar Kyoto rats (NWKR) and stroke prone spontaneously hypertensive rats (SPSHR) were reported [92]. Astaxanthin was found in the plasma, heart, liver, platelets, and increased basal arterial blood flow in mice fed with astaxanthin derivative [93]. Human umbilical vien endothelial cells and platelets treated with the astaxanthin showed increased nitric oxide levels and decrease in peroxynitrite levels [93]. Mice fed 0.08% astaxanthin had higher heart mitochondrial membrane potential and contractility index compared to the control group [94]. Astaxanthin effects on paraoxonase, thioredoxin reductase activities, oxidative stress parameters and lipid profile in hypercholesterolemic rabbits were evaluated. Astaxanthin prevented the activities of those enzymes from hypercholesterolemia induced protein oxidation at the dosages of 100 mg and 500 mg/100 g [67].

### 8.6. Anticancer Activity

The specific antioxidant dose may be helpful for the early detection of various degenerative disorders. Reactive oxygen species such as superoxide, hydrogen peroxide and hydroxyl radical are generated in normal aerobic metabolism. Singlet oxygen is generated by photochemical events whereas peroxyl radicals are produced by lipid peroxidation. These oxidants contribute to aging and degenerative diseases such as cancer and atherosclerosis through oxidation of DNA, proteins and lipids [95]. Antioxidant compounds decrease mutagenesis and carcinogenesis by inhibiting oxidative damage to cells. Cell–cell communication through gap junctions is lacking in human tumors and its restoration tends to decrease tumor cell proliferation. Gap junctional communication occurs due to an increase in the connexin-43 protein via upregulation of the connexin-43 gene. Gap junctional communication was improved in between the cells by natural carotenoids and retinoids [96]. Canthaxanthin and astaxanthin derivatives enhanced gap junctional communication between mouse embryo fibroblasts [97,98,99]. Increased connexin-43 expression in murine fibroblast cells by β-carotene was reported [100,101]. Astaxanthin showed significant antitumor activity when compared to other carotenoids like canthaxanthin and β-carotene [102,103]. It also inhibited the growth of fibrosarcoma, breast, and prostate cancer cells and embryonic fibroblasts [104]. Increased gap junctional intercellular communication in primary human skin fibroblasts cells were observed when treated with astaxanthin [99]. Astaxanthin inhibited cell death, cell proliferation and mammary tumors in chemically induced male/female rats and mice [105,106,107,108,109]. *H. pluvialis* extract inhibited the growth of human colon cancer cells by arresting cell cycle progression and promoting apoptosis reported by Palozza *et al.* [104]. Nitroastaxanthin and 15-nitroastaxanthin are the products of astaxanthin with peroxynitrite, 15-nitroastaxanthin anticancer properties were evaluated in a mouse model. Epstein-Barr virus and carcinogenesis in mouse skin papillomas were significantly inhibited by astaxanthin treatment [110].

### 8.7. Immuno-Modulation

Immune system cells are very sensitive to free radical damage. The cell membrane contains poly unsaturated fatty acids (PUFA). Antioxidants in particular astaxanthin offer protection against free radical damage to preserve immune-system defenses. There are reports on astaxanthin and its effect on immunity in animals under laboratory conditions however clinical research is lacking in humans. Astaxanthin showed higher immuno-modulating effects in mouse model when compared to β-carotene [111]. Enhanced antibody production and decreased humoral immune response in older animals after dietary supplementation of astaxanthin was reported [111,112]. Astaxanthin produced immunoglobulins in human cells in a laboratory study [113]. Eight week-supplementation of astaxanthin in humans [72] resulted in increased blood levels of astaxanthin and improved activity of natural killer cells which targeted and destroyed cells infected with viruses. In this study, T and B cells were increased, DNA damage was low, and C-reactive protein (CRP) was significantly lower in the astaxanthin supplemented group [67,102,114]. Recent reports on astaxanthin biological activities are presented in Table 2.

**Table 2:** Astaxanthin biological activities in *in vitro* and *in vivo* models.

Biological Activities	References
Antioxidant activity	[14,15,17,115,116,117,118,119,120]
Protection from UV rays	[14]
Anti-skin cancer	[14,110,121]
Anti-inflammatory	[84,122,123,124,125]
Anti-gastric activity	[68,71]
Anti-hepatoprotective	[126]
Anti-diabetes	[90,127,128]
Cardiovascular prevention	[94,122,129,130]
Immune response	[72,114]
Neuroprotection	[131,132]

## 9. Safety and Dose of Astaxanthin

Astaxanthin is safe, with no side effects when it is consumed with food. It is lipid soluble, accumulates in animal tissues after feeding of astaxanthin to rats and no toxic effects were found [15,17,133]. Excessive astaxanthin consumption leads to yellow to reddish pigmentation of the skin in animals. Astaxanthin is incorporated into fish feed, resulting in the fish skin becoming reddish in color. Antioxidant enzymes such as superoxide dismutase, catalase, and glutathione peroxidase levels significantly increased in rats after oral dosage of astaxanthin [14,15]. A study reported that blood pressure (bp) was reduced in stroke prone rats and in hypertensive rats by feeding 50 mg/kg astaxanthin for five weeks and 14 days, respectively [134]. Astaxanthin was also shown significant protection against naproxen induced gastric, antral ulcer and inhibited lipid peroxidation levels in gastric mucosa [67,135]. Astaxanthin accumulation in eyes was observed when astaxanthin was fed to rats [136]. Astaxanthin extracted from *Paracoccus carotinifaciens* showed potential antioxidant and also anti-ulcer properties in murine models as reported by Murata *et al.* [137]. Astaxanthin bioavailability was increased with supplement of lipid based formulations [14,15,17,138]. Supratherapeutic concentrations of astaxanthin had no adverse effects on platelet, coagulation and fibrinolytic function [139]. Research has so far reported no significant side effects of astaxanthin consumption in animals and humans. These results support the safety of astaxanthin for future clinical studies.

It is recommended to administer astaxanthin with omega-3 rich seed oils such as chia, flaxseed, fish, nutella, walnuts and almonds. The combination of astaxanthin (4–8 mg) with foods, soft gels and capsules and cream is available in the market. Recommended dose of astaxanthin is 2–4 mg/day. A study reported that no adverse effects were found with the administration of astaxanthin (6 mg/day) in adult human subjects [140]. Astaxanthin effects on human blood rheology were investigated in adult men subjects with a single-blind method after administration of astaxanthin at 6 mg/day for 10 days [141]. Recent studies on astaxanthin dosage effects on human health benefits were presented in Table 3.

**Table 3:** Health benefits of astaxanthin in human subjects.

Duration of Experiment	Subjects in Humans	Dosage (mg/day)	Benefits of Astaxanthin	References
2 weeks	Volunteers	1.8, 3.6, 14.4 and 21.6	Reduction of LDL oxidation	[21]
Single dose	Middle aged male volunteers	100	Astaxanthin take up by VLDL chylomicrons	[60]
8 weeks	Healthy females	0.2 and 8	Decreased plasma 8-hydoxy-2′-deoxyguanosine and lowered in CRP levels	[72]
8 weeks	Healthy adults	6	Assessed by blood pressure	[140]
10 days	Healthy males	6	Improved blood rheology	[141]
12 weeks	Healthy non-smoking finnish males	8	Decreased oxidation of fatty acids	[142]
12 months	Age related macular degeneration	4	Improved central retinal dysfunction in age related macular degeneration	[143]
12 weeks	Middle aged/elderly	12	Improved Cog health battery scores	[144]
12 weeks	Middle aged/elderly	6	Improved groton maze learning test scores	[144]
8 or 6 weeks	Healthy female or male	6	Improved skin winkle, corneocyte layer, epidermis and dermis	[145]
2 weeks	Disease (bilateral cataract)	6	Improved superoxide scavenging activity and lowered hydroperoxides in the human aqueous humor	[146]

LDL, Low-density lipoproteins, VLDL, Very low-density lipoprotein, CRP, C-reactive protein.

## 10. Commercial Applications of Astaxanthin

In the present scenario, production of astaxanthin from natural sources has become one of the most successful activities in biotechnology. Astaxanthin has great demand in food, feed, nutraceutical and pharmaceutical applications. This has promoted major efforts to improve astaxanthin production from biological sources instead of synthetic ones. According to the current literature, astaxanthin is used in various commercial applications in the market. Astaxanthin products are available in the form of capsule, soft gel, tablet, powder, biomass, cream, energy drink, oil and extract in the market (Table 4). Some of the astaxanthin products were made with combination of other carotenoids, multivitamins, herbal extracts and omega-3, 6 fatty acids. Patent applications are available on astaxanthin for preventing bacterial infection, inflammation, vascular failure, cancer, cardiovascular diseases, inhibiting lipid peroxidation, reducing cell damage and body fat, and improving brain function and skin thickness (Table 5). Astaxanthin containing microorganisms or animals find many applications in a wide range of commercial activities, the reason for which astaxanthin enriched microalgae production can provide more attractive benefits.

**Table 4:** Astaxanthin products from various companies and its use for various purposes.

Brand Name	Dosage form	Ingredients	Company Name	Purpose
Physician Formulas	Soft gel/Tablets	2 mg/4 mg-AX	Physician formulas vitamin company	Antioxidant
Eyesight R_x_	Tablet	AX, vitamin-C, plant extracts	Physician formulas Vitamin company	Vision function
KriaXanthin	Soft gel	1.5 mg-AX, EPA, DHA	Physician formulas vitamin company	Antioxidant
Astaxanthin Ultra	Soft gel	4 mg-AX	AOR	Cardiovascular health/gastrointestinal
Astaxanthin Gold™	Soft gel	4 mg-AX	Nutrigold	Eye/joint/skin/immune health
Best Astaxanthin	Soft gel	6 mg-AX, CX	Bioastin	Cell membrane/blood flow
Dr.Mercola	Capsules	4 mg AX, 325 mg Omega-3 ALA	Dr. Mercola premium supplements	Aging/muscle
Solgar	Soft gel	5 mg-AX	Solgar global manufacture	Healthy skin
Astaxanthin	Cream	AX, herbal extracts	True botanica	Face moisturizing
astavita ex	Capsules	8 mg AX, T3	Fuji Chemical Industry	Agingcare
astavita SPORT	Capsules	9 mg AX, T3 and zinc	Fuji Chemical Industry	Sports nutrition
AstaREAL	Oil, powder, water soluble, biomass	AX, AX-esters	Fuji Chemical Industry	Soft gel, tablet, beverages, animal feed, capsules
AstaTROL	Oil	AX	Fuji Chemical Industry	Cosmetics
AstaFX	Capsules	AX	Purity and products evidence based nutritional supplements	Skin/cardiovascular function
Pure Encapsulations	Capsules	AX	Synergistic nutrition	Antioxidant
Zanthin Xp-3	Soft gel capsules	2 mg, 4 mg-AX	Valensa	Human body
Micro Algae Super Food	Soft gel	4 mg AX	Anumed intel biomed company	heart/eye/joint

(Information obtained from the respective company websites); AX, astaxanthin, AXE, astaxanthin esters, CX, canthaxanthin, DHA, docosahexaenoic acid, EPA, eicosapentaenoic acid, ALA, alpha linolenic acid, T3, tocotrienol.

**Table 5:** Recent patent applications for astaxanthin.

Patent No.	Title	Purpose	References
US20060217445	Natural astaxanthin extract reduces DNA oxidation	Reduce endogenous oxidative damage	[147]
US20070293568	Neurocyte protective agent	Neuroprotection	[148]
US20080234521	Crystal forms of astaxanthin	Nutritional dosage	[149]
US20080293679	Use of carotenoids and carotenoid derivatives analogs for reduction/ inhibition of certain negative effects of COX inhibitors	Inhibit of lipid peroxidation	[150]
US20090047304	Composition for body fat reduction	Inhibits body fat	[151]
US20090069417	Carotenoid oxidation products as chemopreventive and chemotherapeutic agents	Cancer prevention	[152]
US20090136469	Formulation for oral administration with beneficial effects on the cardiovascular system	Cardiovascular protection	[153]
US20090142431	Algal and algal extract dietary supplement composition	Dietary supplement	[154]
US20090297492	Method for improving cognitive performance	Improving brain function	[155]
US20100158984	Encapsulates	Capsules	[156]
US20100204523	Method of preventing discoloration of carotenoid pigment and container used therefor	Prevention of discoloration	[157]
US20100267838	Pulverulent carotenoid preparation for colouring drinks	Drinks	[158]
US20100291053	Inflammatory disease treatment	Preventing inflammatory disease	[159]
US20120004297	Agent for alleviating vascular failure	Preventing vascular failure	[160]
US20120114823	Feed additive for improved pigment retention	Fish feed	[161]
US20120238522	Carotenoid containing compositions and methods	Preventing bacterial infections	[162]
US20120253078	Agent for improving carcass performance in finishing hogs	Food supplements	[163]
US20130004582	Composition and method to alleviate joint pain	Reduced joint pain and symptoms of osteoarthritis	[164]
US20130108764	Baked food produced from astaxanthin containing dough	Astaxanthin used in baked food	[165]

## 11. Conclusion

The current research data on astaxanthin is encouraging and have resulted from well controlled trials in *in vitro* and *in vivo* models. Astaxanthin showed potential effects on various diseases including cancers, hypertension, diabetes, cardiovascular, gastrointestinal, liver, neurodegenerative, and skin diseases. Its antioxidant properties are used against oxidative damage in diseased cells. Recently, our laboratory isolated and characterized astaxanthin and its esters from *Haematococcus* and checked their biological activities in *in vitro* and *in vivo* models, confirming that astaxanthin and its esters show potential biological activities in animal models. However, there is a lack of research on astaxanthin esters (mono-di) and their metabolic pathways in biological systems. Future research should focus on effects of astaxanthin esters on various biological activities and their uses in nutraceutical and pharmaceutical applications. Astaxanthin mono-diesters may increase biological activities better than the free form which can be easily absorbed into the metabolism. Further research requires to be investigated on their metabolic pathways and also molecular studies in *in vitro* and *in vivo* models for their use in commercial purposes.
